# Disability in children and adolescents: the extent of the impact on psychiatric disorders and educational deficits

**DOI:** 10.47626/2237-6089-2020-0059

**Published:** 2021-10-22

**Authors:** Livia Zaqueu, Maria Crista Triguero Veloz Teixeira, Rosane Lowenthal, Jair J. Mari, Eurípedes Constantino Miguel, Luís Augusto Rohde, Cristiane Silvestre Paula

**Affiliations:** 1 Programa de Pós-Graduação em Gestão de Ensino da Educação Básica Universidade Federal do Maranhão São Luís MA Brazil Programa de Pós-Graduação em Gestão de Ensino da Educação Básica, Universidade Federal do Maranhão (UFMA), São Luís, MA, Brazil.; 2 Programa de Pós-Graduação em Distúrbios do Desenvolvimento Universidade Presbiteriana Mackenzie São Paulo SP Brazil Programa de Pós-Graduação em Distúrbios do Desenvolvimento, Universidade Presbiteriana Mackenzie (UPM), São Paulo, SP, Brazil.; 3 Departamento de Saúde Mental Faculdade de Ciências Médicas Santa Casa de São Paulo São Paulo SP Brazil Departamento de Saúde Mental, Faculdade de Ciências Médicas da Santa Casa de São Paulo, São Paulo, SP, Brazil.; 4 Departamento de Psiquiatria Universidade Federal de São Paulo São Paulo SP Brazil Departamento de Psiquiatria, Universidade Federal de São Paulo (UNIFESP), São Paulo, SP, Brazil.; 5 Departamento de Psiquiatria Faculdade de Medicina Universidade de São Paulo São Paulo SP Brazil Departamento de Psiquiatria, Faculdade de Medicina, Universidade de São Paulo (USP), São Paulo, SP, Brazil.; 6 Departamento de Psiquiatria Universidade Federal do Rio Grande do Sul Porto Alegre RS Brazil Departamento de Psiquiatria, Universidade Federal do Rio Grande do Sul (URGS), Porto Alegre, RS, Brazil.

**Keywords:** Disability, child psychiatry, adolescent health, epidemiology

## Abstract

**Introduction:**

Most children/adolescents with disability live in low and middle-income countries and, worldwide, they are more likely to have mental health problems and achieve worse academic performance compared to those with typical development.

**Objective:**

To assess whether Brazilian children/adolescents with four types of disabilities are more likely to have psychiatric disorders and educational deficits than children/adolescents with typical development.

**Method:**

A multicenter cross-sectional study involving a school-based probabilistic sample of second to sixth graders (N = 1,674) from public schools in four Brazilian regions. The four types of disabilities (intellectual, visual, hearing, and motor) were assessed using the Ten Questions Questionnaire. Psychiatric disorders were measured with the Schedule for Affective Disorders/Schizophrenia for School-Age Children (K-SADS-PL), and academic performance was evaluated using the Teste de Desempenho Acadêmico – TDE (the academic performance test).

**Results:**

A logistic regression model with cluster-robust errors identified the following statistically significant associations with three of the four types of disability (the exception was hearing). Intellectual disability was associated with anxiety (p < 0.01), depression (p < 0.01), attention deficit hyperactivity disorder (ADHD) (p < 0.001), school failure (p < 0.01), and poor academic performance (p < 0.01). Visual disability was associated with depression (p < 0.01). Motor disability was marginally associated with ADHD (p = 0.08).

**Conclusions:**

Presence of disabilities (intellectual, visual, and motor) in children/adolescents was associated with psychiatric disorders, school failure, and academic performance. It is therefore important to identify presence of disabilities and plan and deliver specific interventions and specialized educational care for the needs presented by these children/adolescents. This is particularly important in low and middle-income countries, where these disabilities are frequent among children/adolescents.

## Introduction

Approximately 200 million children/adolescents around the world have some type of disability, the majority of whom live in low and middle-income countries (LMICs). Children/adolescents with disabilities seem to be more likely to have psychiatric disorders (PD) compared to those with typical development,^1^ but data on the prevalence rates of PD in children/adolescents with disability in Brazil are still scarce.^2^

Worldwide, disabilities and PD are conditions that are individually often associated with worse academic performance during childhood and their co-occurrence tends to impact performance even more negatively,^3^ highlighting the need for priority actions addressed to this group. However, there are few studies in LMICs (including Brazil) that examine this comorbidity or its association with academic performance based on primary data. Therefore, actions in this area are hampered by a lack of reliable and specific data in respect of the Brazilian population.

The objective of this study was, therefore, to investigate the presence of psychiatric disorders and educational deficits among children/adolescents with different types of disabilities, compared with those without disabilities.

## Methods

A multicenter cross-sectional study was carried out with a school-based probabilistic sample from four Brazilian regions (North/Northeast/Center/Southeast). In one town in each region, we randomly selected 500 students from grades 2-6 (ages 6-16 years) from all local public schools. From this initial sample of 2,000 students, 1,674 comprised the final sample (retention rate: 83.7%).

All caregivers signed an informed consent form before participating in the study, which was approved by the Research Ethics Committees (CAAE 16434713.9.3001.5505). Additional information on the methods of the original study can be found in previous publications.^4,5^

### Measurements and variables

The main study outcomes are psychiatric disorders assessed using the Brazilian version of the Kiddie Schedule for Affective Disorders and Schizophrenia – Present and Lifetime version (K-SADS-PL). According to the mother’s/main caregiver’s responses to the K-SADS-PL, presence of a psychiatric disorder was defined as the occurrence of one or more of the four most prevalent PD: depression, anxiety, attention deficit hyperactivity disorder (ADHD), and conduct disorder/oppositional defiant disorder.

Educational deficits (secondary outcomes) were defined as: (1) school failure (maternal report), and (2) academic performance (using the Academic Performance Test [Teste de Desempenho Escolar – TDE] – an instrument developed in Brazil to assess academic performance [reading/writing/arithmetic]).^6^ The TDE classifies children/adolescents into six categories, which we merged into two groups: Middle-low/low/borderline versus Middle/middle-high/high.

The main predictors were four indicators of disabilities assessed using the Ten Question Questionnaire (TQQ), a ten-question instrument that assesses functional limitations in the domains speech, cognition (intellectual disabilities-ID), hearing, and vision. Although the TQQ was developed for children younger than 9, a TQQ unidimensional solution showed good fit indices in our sample, with almost no discrimination in score distribution among children aged 6-9 years compared with those aged 10-16 years, similar to findings of other studies (0.60-0.66).

### Data analysis

Bivariate analyses were conducted and the chi-square test was used to evaluate the statistical significance of the odds ratios (OR) and respective 95% confidence intervals (95%CI) estimated to measure associations between dependent and independent variables. Multi-level logistic regression models were fitted to simultaneously evaluate the effects of the four types of disabilities on each dependent variable (the four PD and the two educational deficits), where the effect of the variable city was incorporated in the form of a random effect. These models did not prove to be valid for all outcomes, and did not converge for depressive disorder. Therefore, new logistic regression models were generated with cluster-robust errors, not including the variable “city.” These models allowed data to be analyzed independent of the cities in which children/adolescents lived.

Gender, age, and social class were used as control variables. All of the explanatory variables were included in the initial multivariate models and were then excluded one by one according to their statistical significance. The final models included all significant (p < 0.05) and marginally significant (0.06-0.10) variables. The goodness-of-fit of the final model was assessed using the Hosmer-Lemeshow test.

## Results

This sample comprised children/adolescents aged from 6 to 16 (53.1% were aged 6-9 years), 52.7% were male, and the majority belonged to middle and lower-middle class families (85.8%). There were high rates of school failure (35.2%). The rates of disabilities and psychiatric disorders have been published previously^7,8^ and are available in Table S1, available as online-only supplementary material.

Bivariate and multivariate analyses were conducted to better understand the simultaneous influence of speech, cognition, hearing, and visual disabilities on depression, anxiety, conduct disorder, and ADHD, as well as on educational deficits (school failure and academic performance), controlling for gender, age, and social class. The same models were adopted to examine the concurrent effects of the four indicators of disabilities and the four psychiatric disorders on the two educational deficits.

Compared to children/adolescents without ID, children/adolescents with ID were more likely to be diagnosed with anxiety (p < 0.01; 95%CI 1.46-5.68), depression (p < 0.01; 95%CI 1.71-32.01), and ADHD (p < 0.01; 95%CI 3.54-4.76) and also had worse academic performance (p < 0.01; 95%CI 1.19-2.66) and a higher rate of school failure (p < 0.01; 95%CI 2.38-3.77).

Compared to their non-visually impaired peers, children/adolescents with visual impairment were more likely to have depression (p < 0.01; 95%CI 1.33-5.59). No factors were statistically associated with having a hearing disability. Having a motor disability was marginally associated with ADHD (p = 0.08; 95%CI 0.95-2.98).

In addition, depression was strongly and independently associated with a higher risk of school failure (OR: 6.41; 95%CI 2.27-18.16), while anxiety was weakly and independently (small effect size) associated with better academic performance (OR: 0.87; 95%CI 0.79-0.96). Table 1 shows the findings of the multivariate analysis.

**Table 1:** Multiple logistic regression and odds ratios for the psychiatric disorders (anxiety, depression, conduct/oppositional defiant disorder, and ADHD) and educational indicators according to four disability indicators (intellectual disability/visual disability/hearing disability/motor disability) (N = 1,674)

Outcomes/factors*	OR_**Crude**_	OR_**Adjusted**_^†^	95%CI for OR_**Adjusted**_	p-value^**‡**^
Anxiety				
Intellectual disability	3.20	2.88	(1.46-5.68)	0.002
Visual disability	1.21	1.03	(0.79-1.34)	0.833
Hearing disability	1.80	1.35	(0.55-3.35)	0.516
Motor disability	1.89	1.36	(0.85-2.17)	0.203
Depression				
Intellectual disability	9.15	7.40	(1.71-32.01)	0.007
Visual disability	3.43	2.72	(1.33-5.59)	0.006
Hearing disability	2.05	1.18	(0.23-6.12)	0.842
Motor disability	0.00	1.00	-^§^	-^§^
				
Conduct/oppositional defiant disorder				
Intellectual disability	1.76	1.40	(0.88-2.23)	0.158
Visual disability	1.14	1.14	(0.51-2.51)	0.751
Hearing disability	1.81	1.51	(0.64-3.59)	0.348
Motor disability	1.80	1.46	(0.40-5.29)	0.564
Attention deficit hyperactivity disorder				
Intellectual disability	4.80	4.10	(3.54-4.76)	< 0.001
Visual disability	1.21	1.05	(0.54-2.07)	0.881
Hearing disability	2.27	1.47	(0.85-2.55)	0.167
Motor disability	2.68	1.68	(0.95-2.98)	0.076
School failure				
Intellectual disability	3.20	2.99	(2.38-3.77)	< 0.001
Visual disability	1.33	1.04	(0.70-1.54)	0.855
Hearing disability	1.68	1.09	(0.64-1.84)	0.761
Motor disability	2.18	1.45	(0.91-2.30)	0.120
Anxiety	1.64	1.21	(0.66-2.23)	0.538
Depression	12.97	6.41	(2.27-18.16)	< 0.001
Conduct/oppositional defiant disorder	2.63	1.70	(0.87-3.29)	0.119
Attention deficit hyperactivity disorder	2.47	1.42	(0.80-2.53)	0.227
Academic performance – inferior				
Intellectual disability	1.86	1.78	(1.19-2.66)	0.005
Visual disability	0.99	0.79	(0.54-1.13)	0.197
Hearing disability	0.98	0.68	(0.30-1.57)	0.369
Motor disability	1.60	1.20	(0.85-1.69)	0.299
Anxiety	1.05	0.87	(0.79-0.96)	0.007
Depression	2.85	1.39	(0.36-5.42)	0.637
Conduct /oppositional defiant disorder	1.69	1.25	(0.46-3.34)	0.663
Attention deficit hyperactivity disorder	1.49	1.12	(0.41-3.06)	0.827

95%CI = 95% confidence interval; OR = odds ratio.

* For all factors, the category absent was adopted as reference.

^†^ Odds ratios adjusted by sex, age, and social class.

^‡^ p-value according to the Wald test for multiple logistic regression.

^§^ No cases of motor disability.

## Discussion

Our findings demonstrated significant associations between the different types of psychiatric disorders (except conduct/oppositional defiant disorder) and three out of the four types of disabilities assessed (the exception being hearing). The strongest associations were with cognitive impairment. These findings show that the associations between psychiatric disorders and comorbid disabilities are complex. Previous studies have drawn attention to the difficulty of identifying comorbid cases, often resulting in late diagnosis and delayed interventions.^9^

As expected, the results showed significant associations between ID and low academic performance and a higher risk of school failure, corroborating previous results.^10^ Experts have recommended using assistive technology resources that allow adaptations to learning tasks and changing the school syllabus according to the specific characteristics of the student.^11^ Moreover, a higher prevalence of anxiety, depression and ADHD was identified among children/adolescents with ID, in line with earlier studies.^1^ These results emphasize that identification of/care for psychiatric symptoms has been neglected in this population, on the assumption that they would be intrinsically associated with ID, however they must be specifically treated to promote adaptive functioning, especially in the school context,^12^ and there should be increased education and skills training for professionals and parents.^13^

In respect of the visually impaired children/adolescents, our data showed a higher risk of depression when compared to their peers without visual impairment. Sensorial limitations and blindness have been described as factors associated with emotional problems and psychiatric disorders. A recent systematic review about rehabilitation programs for visually impaired students indicated promising results related to depression and highlighted the need for training for teachers to be able to better identify and refer comorbid cases.^14^

Among the same sample, motor disability was marginally associated with ADHD. Although there is insufficient information to explain this association, there seems to be an overlap in symptoms, with some individuals with ADHD presenting deficits in fine and gross motor coordination, global kinetic movements, global motricity, balance, body schema, and spatial and temporal organization.^15,16^

Finally, depression was independently associated with a higher risk of school failure, probably because PD can affect different adaptive indicators, including academic performance.^17^ The association observed between anxiety and better academic performance in our study was surprising and must be interpreted with caution due to its small effect size.^18^ Anxiety is usually associated with reduced school achievement, however, the effects of emotions on achievement are inevitably complex and in some cases low-level anxiety is associated with increased motivation and better results.^17^

To the best of our knowledge, this is the first Brazilian multicenter study exploring these associations, but some limitations must be mentioned: (1) there was no confirmation of the diagnosis of the four disabilities after the screening stage; (2) the disability indicators were collected using mothers’ reports, without clinical evaluation of the child; (3) the TQQ questions are generalist, and may bias the answers; (4) although they are a minority group, children/adolescents outside the school system or attending private schools were not included in the study, which limits the generalizibiliy of the results; and (5) since this was a cross- sectional study, we cannot guarantee the direction of causality, but because disability tends to have earlier onset, this, at least partly, minimizes the problem of reverse causality.

Despite these limitations, the study contributes new data to the field, demonstrating that it is possible to trace indicators of disabilities in the school environment, as well as to identify groups with greater vulnerability. This quick and cost-effective strategy can be used as a model that can be scaled-up, helping to make more accurate referrals to health and education systems.

In conclusion, our data shows a high co-occurrence of disabilities and psychiatric disorders (mainly depression and anxiety) with associated academic impacts in students from different regions of the country. Thus, educational and health services need to be prepared to offer differentiated care to these comorbid cases.
