# Supplementary material for: Disability in children and adolescents: the extent of the impact on psychiatric disorders and educational deficits
Source: Trends Psychiatry Psychother. 2021 Oct 22;43(3):235–9. doi: 10.47626/2237-6089-2020-0059 (PMC8638709; doi:10.47626/2237-6089-2020-0059)
Supplement: Supplementary file 1 [file 2238-0019-trends-43-03-0235-suppl01.pdf]

http://dx.doi.org/10.47626/2237-6089-2020-0059

Table S1 - Frequency of disabilities (intellectual, visual, hearing, and motor) and psychiatric disorders (anxiety, depression, conduct/oppositional defiant disorder, and ADHD), N = 1,674

	**N**	**% positive**
Disabilities		
Intellectual	415	24.8
Visual	252	15.1
Hearing	109	6.5
Motor	141	8.4
Psychiatric disorders		
Anxiety*	113	6.9
Depression†	8	0.5
Conduct /oppositional defiant disorder‡	36	2.2
ADHD§	69	4.1

ADHD = attention deficit hyperactivity disorder.
* 25 missing.
† 9 missing.
‡ 31 missing.
§ 10 missing.
